# Dual-core silver-coated plasmonic sensor modeling with machine learning

**DOI:** 10.1016/j.heliyon.2024.e38175

**Published:** 2024-09-23

**Authors:** Chanchal Saha, Farzana Haque, Nazrul Islam, Muhammad Minoar Hossain, Md. Easin Arafat, Mohammad Abu Yousuf, Mohammad Motiur Rahman

**Affiliations:** a Department of Computer Science and Engineering, Mawlana Bhashani Science and Technology University, Tangail-1902, Bangladesh; b Institute of Information Technology, Jahangirnagar University, Savar, Dhaka-1342, Bangladesh; c Department of Information and Communication Technology, Mawlana Bhashani Science and Technology University, Tangail-1902, Bangladesh

**Keywords:** Surface plasmon resonance, Plasmonic sensor, Machine learning, Dual-core silver-coated, PCF-SPR sensor

## Abstract

Plasmonic sensors utilizing surface plasmon resonance (SPR) have emerged as powerful tools for sensitive and label-free detection across a wide range of applications. This study introduces a new dual-core silver-coated plasmonic sensor designed to significantly enhance sensitivity and resolution, making it particularly effective for precise analyte detection in complex environments. A key innovation of this sensor lies in its dual-core architecture, which achieves the highest wavelength sensitivity reported at 30,000 nm/RIU and resolution of $3.33\times 10^{-6}$ RIU, covering a broad refractive index (RI) range from 1.34 to 1.41. Furthermore, the integration of machine learning (ML) algorithms, including multiple-variable linear regression (MLR), support vector regression (SVR), and random forest regression (RFR), marks a significant advancement in sensor design. These algorithms enable dynamic adaptation and the extraction of data-driven insights, enhancing the sensor's performance in predicting confinement loss and wavelength across various analytes. The innovative combination of a dual-core design and ML integration positions this plasmonic sensor as a highly sensitive and versatile tool well-suited for advanced bio-sensing applications.

## 1 Introduction

Plasmonic sensors, using the unique light properties of surface plasmons, have become solid tools for susceptible and label-free detection in many fields. Combining ML techniques with plasmonic sensing is an excellent way to make sensors work even better and be helpful in more situations. This teamwork helps get important information from complicated sensor data, making sensors better at noticing things, choosing what to see, and keeping track of changes quickly. The new dual-core silver-coated plasmonic sensor has many advantages over old single-core ones. Adding two cores makes the sensor more sensitive and robust, so it can find many things at once or notice different things better. With ML helping out, these improvements make sensors much more accurate and trustworthy for finding things. Integrating ML with plasmonic sensor technology has propelled a significant breakthrough across numerous applications, especially in domains demanding empathetic detection capabilities. Plasmonic sensors, acclaimed for their proficiency in enhancing optical signals at the nanoscale and the predictive and analytical strength of ML, have unveiled new possibilities in diagnostics [1], pollutant detection [2], and the study of materials [3].

Recently, photonics technology has also improved by incorporating ML techniques [4], surpassing traditional photonics in efficiency and performance. Consequently, researchers have shifted their focus towards ML, applying it in various applications such as coherent optical communication systems [5], computing optical properties [6], [7], predicting confinement loss [8], [9] and predicting RI through the application of deep learning techniques [10]. Moreover, the omnidirectional bending sensor, utilizing cascaded asymmetric dual-core PCF in conjunction with ML, delivers exact predictions of curvature and bending orientation across a full 360° range. The sensor achieves remarkable accuracy, with a 99.85% success rate in measuring bending direction and 98.08% in curvature measurement [11].

Furthermore, the authors [12] proposed a highly responsive SPR sensor seamlessly integrated into a dual-core PCF and enhanced with silver and titanium dioxide coatings, which is meticulously optimized for detecting low RI. By leveraging artificial neural networks (ANN) and genetic algorithms, the sensor attains high sensitivity, positioning it as an ideal tool for applications such as pharmaceutical inspection. Similarly, a deep learning-assisted approach utilizing recurrent neural networks (RNN) has been developed to effectively model and predict the figure of merit (FOM) for optimizing fiber optic sensor performance. This approach achieves rapid and accurate predictions, significantly reducing processing time compared to traditional methods [13]. Besides, the study [14] showcases the effectiveness of combining an ML model with particle swarm optimization (PSO) to design a tunable SPR sensor, achieving rapid optimization with a sensitivity of 68.754°/RIU and a FOM of 100. This approach accelerates the sensor design process by substituting complex electromagnetic calculations with ML-driven predictions.

Recent research has made significant strides in developing plasmonic sensors with enhanced sensitivity [15], [16], [17]. However, existing approaches for fabricating these sensors often face challenges related to coating layer precision and the complexity of air-hole arrangements. To address these limitations, improving fabrication tolerances, carefully selecting design parameters, and optimizing external sensing methods are essential to enhance sensor efficiency. Developing superior sensor designs requires careful consideration of factors such as air-hole dimensions and sensitivity to wavelength and resolution, which are crucial for achieving better analyte detection. Additionally, the creation of advanced photonic structures for novel applications in optics and integrated photonics presents a growing need for precise parameter tuning. Traditional methods like the finite-difference time-domain approach, while accurate, are often time-consuming. In contrast, ML offers a much faster alternative, significantly accelerating the optimization of design parameters in complex nanostructures. This advancement is vital for enhancing the efficiency of sensing and integrated photonics applications, representing a key innovation over previous methodologies.

The primary contribution of the paper is outlined as follows:

• Designing a dual-core plasmonic sensor that enhances its performance using silver as the plasmonic material.
• Achieving the highest wavelength sensitivity of 30,000 nm/RIU and a resolution of $3.33\times 10^{-6}$ RIU, it operates within an RI range of 1.34 to 1.41.
• Employing ML algorithms, including MLR, SVR, and RFR, to predict confinement loss and wavelength across various analytes.

## 2 Materials and methods

### 2.1 Proposed sensor design

**Figure 1:**
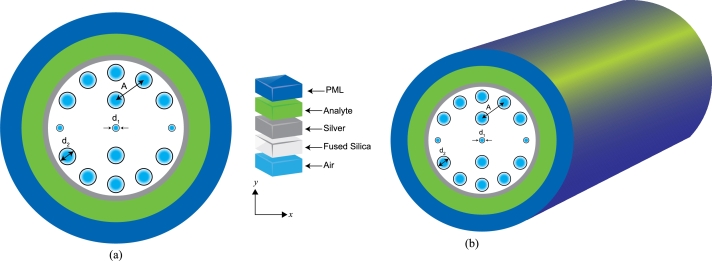
(a) Proposed plasmonic sensor with cross-sectional perspective, (b) 3D view of the sensor.

The proposed sensor is elegantly illustrated in Fig. 1 (a), and a three-dimensional (3D) view is shown in Fig. 1(b). The central element of the sensor comprises a meticulously arranged pattern of air–holes within a fused silica matrix featuring two distinct diameters designated as ($d_{1}$) and ($d_{2}$). The more diminutive air–holes are arrayed around comparatively larger air holes in a regular pattern. Interspersed among these smaller air holes are the larger air holes strategically positioned to adjust the core's effective RI and optimize the coupling of electromagnetic waves into the plasmonic mode. The pitch (*A*) describes the central separation from one air hole's center to the next's center. As a principal geometric determinant, the pitch substantially affects the propagation dynamics of light within the PCF. This, in turn, alters the light's interaction with the plasmonic modes, which is a crucial factor in the sensor's overall sensitivity and detection capability. Enveloping this core configuration is a silver coating, carefully selected for its plasmonic properties. Silver layer ($t_{g}$) is applied to the sensor's outer surface to enhance interaction with guided waves. This metallic layer is intricately engineered to facilitate the excitation of surface plasmon polaritons (SPP) at the interface where the metal meets the dielectric constituents of the fused silica and the analyte. This feature is essential for the sensitivity of the SPR sensor, enabling it to detect minute variations in the RI when the analyte interfaces with the silver layer.

Moreover, the thickness of the silver layer and the dielectric overlay should be adjusted to minimize the coupling strength of higher-order SPP modes. Additionally, select an operational wavelength where the coupling of these higher-order modes is weak or absent. By carefully choosing the wavelength, the impact of these unwanted modes can be effectively minimized. Simulations were performed using the COMSOL Multiphysics simulator (version 5.5) to identify the optimal value for a specific parameter. During these simulations, various values of the parameter in question were tested while keeping all other parameters constant. The optimal parameter selection was guided by the strong coupling observed between the core-guided mode and the SPP mode. After completing the optimization process, the following design parameters were determined: The optimal design includes *A*=1.50 μm, $d_{1}$=0.4 μm, $d_{2}$=0.6 μm, and $t_{g}$=0.03 μm. A unique sensor design has been developed, demonstrating enhanced performance compared to existing sensors referenced in sources [18], [19]. This innovative design allows for more accurate data capture under specific wavelengths, which is crucial for the reliability of the ML predictions.

The perfectly matched layer (PML) represents the schem-atic's outermost boundary. In computational electromagnetic simulations, the PML is essential; it absorbs electromagnetic waves in all directions, effectively eliminating reflections that could distort the simulation results. The PML facilitates accurate sensor characteristics computations without necessitating an impractically extensive simulation space by emulating an infinitely expansive space. The geometric design of the plasmonic sensor, including the precise configuration and dimensions of the air holes alongside the strategic positioning of the silver layer, is meticulously engineered. This design is intended to refine light coupling into the plasmonic mode, thereby enhancing the sensor's susceptibility to RI variations within the analyte. Furthermore, implementing a dual-core architecture featuring small and large air holes augments the interaction between light and analyte, consequently improving the sensor's overall performance. The researchers conducted convergence tests using a customized mesh size and optimized the thickness of the PML. They considered 14,846 triangles, 1,526 edge elements, 76 vertex elements, and 1,04019 degrees of freedom during the computational analysis.

The performance of a dual-core sensor was simulated using COMSOL Multiphysics (version 5.5) and the finite element method (FFM) [20]. The fabrication of this sensor involves creating air holes of different sizes in a silica substrate using the stack-and-draw method [21]. The chemical vapor deposition (CVD) process recently involves a solid material from a vapor by a chemical reaction occurring on or near a heated substrate surface [22]. High-pressure microfluidic chemical deposition [23] and magnetron sputtering coating apply a thin silver layer on the sensor's surface [24]. Hence, the sensor can be efficiently fabricated using existing fabrication technologies.

### 2.2 Mathematical conceptualization

The core material used in the sensor is fused silica, and we employ the Sellmeier equation to calculate the RI suitable for various desired wavelengths using the formula provided below [25].

(1) $$n^{2}(\lambda )=1+\frac{B_{1}\lambda^{2}}{\lambda^{2}-C_{1}}+\frac{B_{2}\lambda^{2}}{\lambda^{2}-C_{2}}+\frac{B_{3}\lambda^{2}}{\lambda^{2}-C_{3}}$$

In this context, ‘n’ represents the RI of pure silica, and ‘*λ*’ represents the wavelength in micrometers. The Sellmeier equation includes coefficients denoted as ‘$B_{1},B_{2},B_{3}$’ and ‘$C_{1},C_{2},C_{3}$’. For fused silica (Si), the specific coefficient values are as follows: $B_{1}=0.69616300$, $B_{2}=0.407942600$, $B_{3}=0.89779400$, and $C_{1}=0.00467914826$, $C_{2}=0.0135120631$, $C_{3}=97.9340025$.

The primary purpose of incorporating silver into SPR-based PCF sensors is to generate surface plasmon waves along the silver-coated fiber surface. The material dispersion of silver can be determined using Drude's model [26].

(2) $$\varepsilon_{Ag}=\varepsilon_{\infty }-\frac{\omega_{D}^{2}}{\omega (\omega +j\gamma_{D})}-\frac{\Delta_{\varepsilon }\Omega_{2}^{2}}{(\omega^{2}-\Omega_{2}^{2})+j\Gamma_{j}\omega }$$

In this context, $\varepsilon_{\infty }$ is set at 2.4064, representing the permittivity of the silver layer. The Drude model employs standard constants, where $\omega_{D}$/2*π* is 2214.6 THz, and $\gamma_{D}$/2*π* is 4.8 THz. Additionally, the Lorentz oscillator involves parameters such as $\Omega_{L}$/2*π* equaling 1330.1 THz, $\Gamma_{L}$/2*π* at 620.7 THz, and a Lorentz oscillator strength $\Delta_{\varepsilon }$ of 1.6604.

Confinement loss directly impacts the effectiveness of light transmission in optical fibers. The configuration of the PCF, encompassing the dimensions and arrangement of the air holes, can be meticulously optimized to regulate confinement losses. By adjusting these structural parameters, the loss properties can be finely tuned to meet the precise requirements of various sensing applications. The following formula is employed for confinement loss calculation [27].

(3) $$\alpha (\text{dB/cm})=8.686\times K_{0}\times \text{Im}(n_{\text{eff}})\times 10^{4}$$

where, $\text{Im}(n_{\text{eff}})$ represents the imaginary part of the effective RI, where $K_{0}$ is defined as 2π/λ, indicating the wave number in free space with *λ* denoting the operational wavelength.

Wavelength sensitivity ($S_{\lambda }$) is an essential measurement for assessing the performance of sensors that use the wavelength interrogation technique. The provided equation allows calculating wavelength sensitivity in PCF [28].

(4) $$S_{\lambda }=\frac{\Delta \lambda_{\text{peak}}}{\Delta n_{a}}$$

The symbol $\Delta \lambda_{\text{peak}}$ represents the change in the peak wavelength, and $\Delta n_{a}$ relates to the RI of two adjacent substances.

In PCF sensors, achieving resolution is paramount for precisely detecting and measuring environmental or monitored parameters. The configuration of the PCF core, defined by the strategic placement and sizing of the air holes, critically influences its light-guiding capabilities. An optimally designed core can support single-mode propagation across very short wavelengths, enhancing the resolution by significantly reducing modal dispersion. The provided equation enables the resolution calculation in PCF [29].

(5) $$R(RIU)=\frac{\Delta n_{a}\times \Delta \lambda_{min}}{\Delta \lambda_{peak}}$$

$\Delta n_{a}$ represents the disparity in RI between two neighboring analytes (e.g., $\Delta n_{a}$ = 0.01), $\Delta \lambda_{min}$ signifies the slightest wavelength difference (e.g., $\Delta \lambda_{min}$ = 0.1 nm), and $\Delta \lambda_{peak}$ quantifies the variation in the peak wavelength. These terms measure alterations or deviations in the wavelengths under consideration.

The mean squared error (MSE) is a common evaluation metric used in ML and statistics to evaluate the quality of a model's predictions. It measures the average squared difference between the predicted and actual values for a set of predictions.

(6) $$MSE=\frac{1}{n}\sum_{i=1}^{n}{\left(y_{i}-\overset{¯}{y_{i}}\right)}^{2}$$

where ‘n’ represents the count of observations, $y_{i}$ denotes the actual value for the $i^{th}$ observation, and $\overset{¯}{y_{i}}$ signifies the predicted value for the $i^{th}$ observation.

Root Mean Squared Error (RMSE) is a frequently used evaluation metric in ML, particularly in regression tasks. It quantifies the standard deviation of errors between predicted and actual values. RMSE is derived by taking the square root of the mean squared error (MSE) and is expressed as:

(7) $$RMSE=\sqrt{\frac{1}{n}\sum_{i=1}^{n}{\left(y_{i}-\overset{¯}{y_{i}}\right)}^{2}}$$

Where ‘n’ signifies the count of observations, $y_{i}$ stands for the actual value, and $\overset{¯}{y_{i}}$ denotes the predicted value. RMSE offers an intuitive evaluation metric with units matching the target variable, aiding in model performance comparisons. A smaller RMSE value indicates superior predictive performance. RMSE is frequently utilized alongside other metrics, such as R-squared ($R^{2}$), to assess model performance comprehensively.

Mean absolute error is another standard evaluation metric used in ML and regression problems. It indicates the typical absolute difference between the expected and observed values. MAE is calculated as:

(8) $$MAE=\frac{1}{n}\sum_{i=1}^{n}|y_{i}-\overset{¯}{y_{i}}|$$

where ‘n’ denotes the number of observations, $y_{i}$ is the actual value, and $\overset{¯}{y_{i}}$ is the predicted value.

### 2.3 ML model implementation

#### 2.3.1 Dataset description

Data for this research was obtained through a plasmonic sensor, depicted in Fig. 1. The COMSOL Multiphysics Simulator (version 5.5) was employed to simulate and generate the dataset. Five attributes, namely the RI of the analyte ($n_{a}$), confinement loss (*α*), operating wavelength (*λ*), the real ($Re_{\text{eff}}$), and imaginary (${\text{Im}}_{\text{eff}}$) parts of the RI were gathered to form the dataset. A total of 1580 data for these attributes were used in the dataset, where the RI of the analyte contained eight distinct values ranging from 1.34 to 1.41, and the rest of the attributes had different continuous values. Table 1 shows the attributes of the dataset in detail. Based on the available knowledge, the RI range of 1.34 to 1.41 is considered a typical operating range for this sensor [30], [31].

**Table 1:** Description of the attributes of the dataset.

Attribute	Description	Notation	No. of Data
Refractive index	Changes in the local optical environment	*n*_*a*_	316
Wavelength	The specific light frequency used for detecting changes due to the analyte.	*λ*	316
Real part	The phase change of the light.	*R*_eff_	316
Imaginary Part	The attenuation or absorption of the light.	Im_eff_	316
Confinement Loss	The amount of light energy lost due to interaction with the PCF's microstructure.	*α*	316

#### 2.3.2 System configuration

The algorithms were executed in a Google Colab environment with GPU support. Python 3.8 was used as the programming language. Python's Numpy, Sklearn, Pandas, and Matplotlib libraries were employed for data manipulation.

#### 2.3.3 ML models

Multiple-variable linear regression is a fundamental technique in ML used to predict a continuous target variable by modeling its relationship with one or more independent variables, also known as features [32]. In ML, the equation of multiple linear regression is expressed as:

(9) $$y=b_{0}+b_{1}X_{1}+b_{2}X_{2}+b_{3}X_{3}+................+b_{n}X_{n}$$

In this context, ‘y’ represents the target variable, while $X_{1}$, $X_{2}$, $X_{3}$, and so on up to $X_{n}$ represent the various features. The model includes an intercept term $b_{0}$ and coefficients $b_{1}$, $b_{2}$, $b_{3}$, and so on up to $b_{n}$, which determine the influence of each feature on the prediction.

Random forests, a versatile technique for both classification and regression, employ multiple decision trees [33]. These trees are created by randomly selecting features and data subsets, reducing overfitting and enhancing model generalization. Support Vector Regression (SVR) is an ML technique used for regression analysis, specifically for predicting continuous output values [34].

#### 2.3.4 Workflow of ML

**Figure 2:**
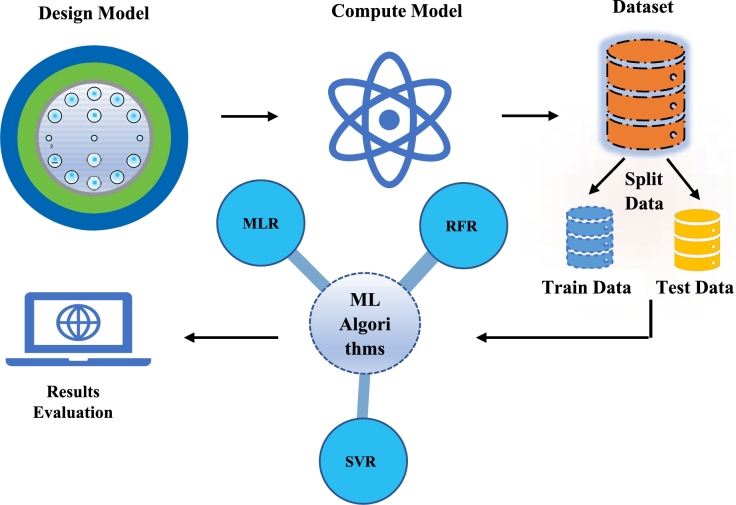
The schematic representation of the proposed workflow of ML.

Fig. 2 depicts a typical workflow for developing and evaluating an ML model. The initial stage in the ML workflow is the design model phase, where the structure and type of the model are defined based on the problem at hand. This involves selecting the appropriate simulator, setting up the model architecture, and determining the features. Decisions at this stage are informed by domain knowledge, research, and preliminary data analysis. The model's design is crucial, laying the foundation for all subsequent steps. The compute model stage involves the computational process of turning the model design into an executable form. This may include setting up computing resources and configuring parameters. The focus here is on the efficiency and scalability of model computation, ensuring that the model can be trained within reasonable time and resource constraints. The dataset is the collection of data used for training and testing the model. It must be well-prepared, cleaned, and formatted to match the model. The quality and quantity of the dataset directly impact the model's performance. Analyzing the dataset involves understanding its distribution, identifying biases, and ensuring it represents the problem domain.

The dataset is divided into training and test data in the split data stage. The training data is used to train the model, while the test data is used to evaluate its performance. The data split should be done carefully to ensure that both sets are representative. Techniques such as cross-validation might be used to make the evaluation more robust.

The ML algorithm is the core component where the actual learning happens. It takes training data to learn patterns and make predictions. Selecting the correct algorithm and tuning its parameters are critical tasks. The choice of algorithm depends on the problem type (classification, regression, clustering, etc.) and the nature of the data. This study used regression. The test data evaluates the model's performance in the results stage. Depending on the problem, this involves predicting the confinement loss and wavelength. Assessing the results helps understand how well the model generalizes to new, unseen data. It also highlights areas where the model might be underperforming, guiding further refinement and iteration. Overall, this workflow illustrates the iterative nature of developing an ML model, emphasizing the importance of each step from model design to results evaluation. Continuously refining and enhancing the model remains the primary objective, aiming for optimal performance.

## 3 Results and analysis

When light travels through the fiber's core, a portion of it extends into the cladding region, forming what is known as the evanescent field. This field interacts with a metal surface, producing a surface plasmon wave (SPW). When the frequencies of the core mode and surface plasmon polariton (SPP) mode match, there is a distinct increase in confinement loss. The generation of SPW is optimized at the resonance wavelength, as evidenced by a sharp peak in the fundamental mode's loss spectrum. A small change in the RI of the sensing medium results in a significant shift in this wavelength, a characteristic used for detecting analytes.

### 3.1 Coupling properties

**Figure 3:**
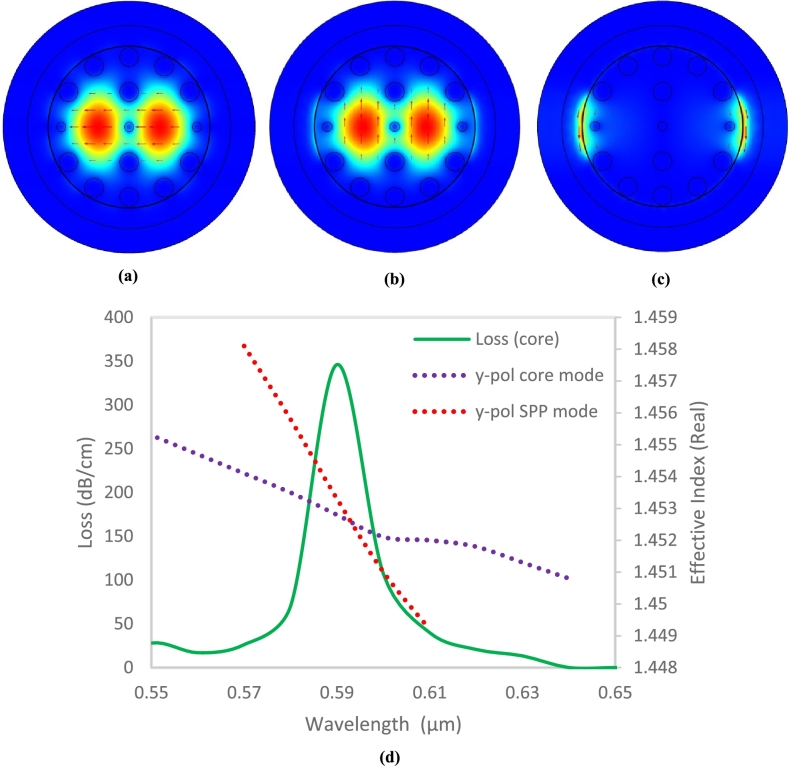
Optical field distribution for (a) x-polarized light, (b) y-polarized light, (c) y-polarized spp mode, (d) determining the phase matching criteria for an analyte with a RI of 1.39 by examining the core and SPP modes along with the loss spectrum for modes polarized in y-axis directions. *A* = 1.50 μm, *d*_1_ = 0.4 μm, *d*_2_ = 0.6 μm and *t*_*g*_ = 0.030 μm, at analyte RI of 1.39.

Fig. 3(a) and 3(b) show the sensor's electric field along with the fundamental polarization of light along the x- and y-axes. Fig. 3(c) displays the y-polarized plasmonic mode, demonstrating the solid guiding characteristics of the carefully structured cladding holes. Due to birefringence, x-polarized and y-polarized modes have differing imaginary parts of the effective RI. In this design, the y-polarized mode has a higher evanescent field due to low core confinement loss, leading to more pronounced coupling. This analysis highlights the significance of y-polarized modes in PCF-based SPR sensors.

Mathematically, SPR occurs when the real part of the effective RI of the sensor's core-guided mode matches that of the SPP mode. At this specific condition, the oscillating surface electrons absorb the maximum energy from the incident light, resulting in a sharp resonance peak in the sensor's loss curve. The unknown sample can be identified by examining this SPR point on the loss curve. Numerical analysis of Fig. 3(d) reveals that this phase-matching condition for the proposed sensor occurs at a wavelength of 0.59 μm for an analyte RI of 1.39. At this resonance wavelength, the real part of the effective RI of the core and SPP modes intersect, leading to a pronounced loss peak. The SPR phenomenon in the sensor can be explained using coupled-mode theory. In an SPR sensor, the coupled-mode equations for the core-guided and SPP modes can be expressed as follows [35].

(10) $$\frac{dE_{1}}{dz}=j\beta_{1}E_{1}+jkE_{2}$$

(11) $$\frac{dE_{2}}{dz}=j\beta_{2}E_{2}+jkE_{1}\sum_{i=1}^{n}X_{i}^{2}$$

$E_{1}$ and $E_{2}$ represent the mode fields for the core-guided and SPP modes, respectively. The terms $\beta_{1}$ and $\beta_{2}$ correspond to the propagation constants of these two modes, while *κ* denotes the coupling strength between them. The variable *z* indicates the propagation length of light.

### 3.2 Performance analysis

**Figure 4:**
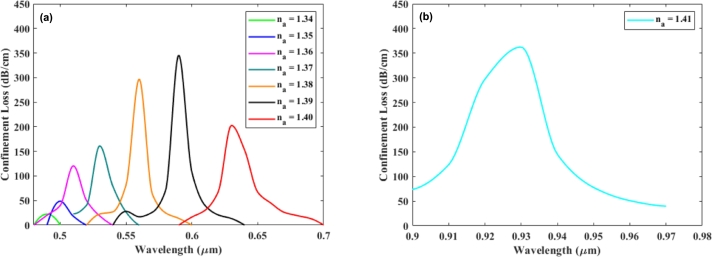
Confinement loss spectrum is analyzed for (a) y-polarized light with an analyte RI (*n*_*a*_) ranging from 1.34 to 1.40, and (b) *n*_*a*_ = 1.41 only.

The peak loss depth consistently increases as the RI of the analyte rises from 1.34 to 1.40 in 0.01 increments, as shown in Fig. 4(a) and the RI of the analyte of 1.41 is shown in Fig. 4(b). The RI contrast between the core and SPP modes diminishes as the analyte's RI increases, leading to a notable shift in confinement loss. Δ$n_{a}$ signifies the sequential analyte RI ($n_{a}$), whereas $\Delta \lambda_{\text{peak}}$ denotes the shift in wavelength at its peak that arises due to variations in the analyte's RI. When the analyte RI undergoes transitions from 1.34 to 1.35, 1.35 to 1.36, 1.36 to 1.37, 1.37 to 1.38, 1.38 to 1.39, and 1.39 to 1.40, the proposed sensor exhibits resonance wavelength shifts of 10, 10, 20, 30, 30, 40, and 300 (nm) in the y-polarized mode. Consequently, as illustrated in Table 2, the corresponding wavelength sensitivities are 1000, 1000, 2000, 3000, 3000, 4000, and a remarkable 30,000 (nm/RIU). The fundamental assumption is that within the y-polarized mode, the sensor achieves resolutions of $1.0\times 10^{-4}$ RIU and $3.33\times 10^{-6}$ RIU at analyte RI of 1.34 and 1.40, respectively. It also shows that as the analyte RI increases, the loss peak in the system also rises, with the lowest confinement loss recorded at 22.36 dB/cm at a wavelength of 0.49 μm for the analyte RI of 1.34 and the highest peak loss of 362.54 dB/cm occurring at 0.93 μm when the analyte RI is 1.41.

**Table 2:** Performance Analysis of silver from analyte 1.34 to 1.41.

*n*_*a*_	*P*_*loss*_	*λ*_*peak*_	Δ*λ*_*peak*_	*S*_*λ*_	*R*
	(dB/cm)	(nm)	(nm)	(nm/RIU)	(RIU)
1.34	22.3627	490	10	1000	1.0 × 10^−4^
1.35	48.602	500	10	1000	1.0 × 10^−4^
1.36	120.5057	510	20	2000	5.0 × 10^−5^
1.37	161.0503	530	30	3000	3.33 × 10^−5^
1.38	297.2436	560	30	3000	3.33 × 10^−5^
1.39	346.1776	590	40	4000	2.50 × 10^−5^
1.40	202.8923	630	300	30000	3.33 × 10^−6^
1.41	362.5364	930	-	-	-

**Figure 5:**
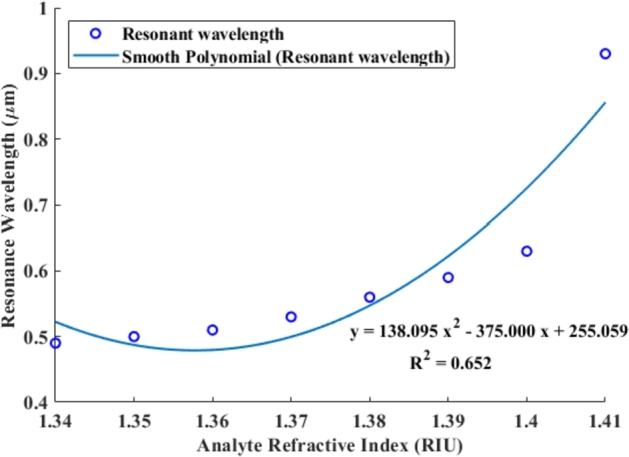
The spectra demonstrate the resonance wavelength scattering linearly correlated with the analyte RI.

The blue line represents a smooth polynomial fit of the data, which is used to model the relationship between the RI and the resonance wavelength, as shown in Fig. 5. The polynomial equation is expressed as

$$y=138.095x^{2}-375.000x+255.059$$

where *y* is the resonance wavelength and *x* is the analyte RI. The R-squared value ($R^{2}=0.652$) indicates the goodness of fit for the polynomial model to the data. An $R^{2}$ value of 0.652 suggests a moderate correlation, implying that the polynomial equation accounts for approximately 65.2% of the resonance wavelength variance based on the RI.

The plot reveals a nonlinear relationship between the analyte RI and the resonance wavelength. Initially, as the RI increases, the resonance wavelength decreases slightly before increasing more rapidly. This behavior is typical of plasmonic sensors, where the resonance condition is highly sensitive to changes in the RI of the surrounding medium due to the interaction of light with surface plasmons. The curve's shape indicates that the sensor's sensitivity to changes in the RI is not constant; it varies with the actual value of the RI. At higher RI values, the rate of change in the resonance wavelength is more pronounced, suggesting greater sensitivity in that range.

**Figure 6:**
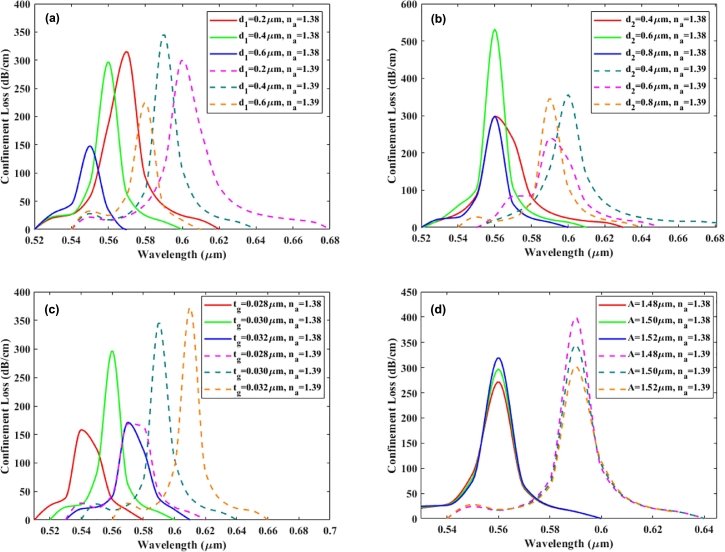
The confinement loss curves are shown for different parameters at analyte RI of 1.38 and 1.39, including (a) small air-holes (*d*_1_), (b) big air-holes (*d*_2_), (c) variations of silver (*t*_*g*_) thickness and (d) pitch variations of the sensor.

Fig. 6(a) portrays the attenuation pattern of the depicted sensor, where the parameter $d_{1}$ experiences a transition in the y-polarized mode, shifting from 0.2 μm to 0.6 μm. It is noteworthy that altering the value of $d_{1}$ significantly influences the attenuation pattern, a fact that can be empirically demonstrated. The attenuation intensity in the y-polarized mode decreases from 315.56 dB/cm to 147.89 dB/cm as the diameter $d_{1}$ increases from 0.2 μm to 0.4 μm for an analyte RI of 1.38. In contrast, for an analyte RI of 1.39, the attenuation intensity initially increases from 300.86 dB/cm to 346.18 dB/cm as $d_{1}$ goes from 0.2 μm to 0.4 μm. It then decreases to 225.09 dB/cm when $d_{1}$ reaches 0.6 μm. Additionally, a slight deviation in resonance is observed with the increase in $d_{1}$. Notably, the y-polarized mode exhibits analogous characteristics in terms of wavelength shifting.

Fig. 6 (b) illustrates the influence of varying the diameter $d_{2}$ of the big air hole on the loss spectrum. The data presented in the graph unequivocally demonstrates that an increase in $d_{2}$ results in a more pronounced loss. Furthermore, as $d_{2}$ is heightened, the discrepancy in the effective RI (neff) between the core and cladding decreases, consequently leading to increased data loss in the presented sensor. Specifically, in the y-polarized mode, when $d_{2}$ is fixed at 0.4 μm, the maximum depth of loss reaches a remarkable 356.19 dB/cm. A comprehensive analysis of the same graph reveals a subtle shift toward a redder hue when $d_{2}$ is augmented. When the diameter is adjusted from 0.4 μm to 0.8 μm, the resonant wavelength transitions from 0.56 to 0.60 μm, and this identical phenomenon is observed in the y-polarized mode.

Changing the sensor's structural features, such as altering the thickness of plasmonic materials, the spacing between them, and the dimensions of air holes, significantly affects how well the sensor works. Nonetheless, the capacity to meticulously preserve these structural attributes throughout the fabrication process is promising. Initially, Fig. 6(c) shows that the silver layer's thickness was altered, transitioning from 0.028 μm to 0.032 μm, and the resultant influence on confinement loss is elucidated in the accompanying diagram. This endeavor was undertaken to probe the ramifications of fine-tuning structural parameters on sensor performance. A thinner silver layer enhances mode confinement and reduces guided mode leakage into nearby cladding or surrounding medium. This implies that increasing the thickness of the silver layer leads to a decrease in confinement loss. Nevertheless, achieving a thin silver coating on the fiber presents a formidable challenge, as a thicker layer curtails the sensor's amplitude. Therefore, an informed decision was made to employ a 0.030 μm silver layer in this configuration.

Fig. 6(d) illustrates that changes in the pitch and the RI of the analyte influence both the position and the height of the confinement loss peaks. This suggests that one can customize the fiber by carefully modifying the pitch and RI to achieve specific confinement loss characteristics. These desired profiles can be targeted at particular wavelengths, offering a level of control for sensor design. Upon closer analysis of the trends, it becomes apparent that keeping the RI constant and gradually increasing the pitch results in a noticeable shift. Specifically, this shift is towards a higher wavelength, where the maximum confinement loss is observed. For instance, with an analyte RI of 1.38, escalating the pitch from 1.48 μm to 1.52 μm results in a discernible transposition of the peak towards the right.

Conversely, holding the pitch constant while modulating the RI induces a similar shift in the peak of confinement loss. As illustrated, with a fixed pitch of 1.50 μm, elevating the RI from 1.38 to 1.39 prompts the peak to migrate to a longer wavelength. The figure highlights the significant impact of both pitch and RI on the confinement loss in a PCF sensor. These two variables alone can dramatically influence the fiber's light-guiding efficiency. This insight is paramount for engineering PCF precisely configured for specific optical properties, facilitating their application in areas like bio-sensing.

Table 3 presents a comparative analysis of the proposed sensor against current plasmonic sensors, examining factors like their structure, RI range, wavelength sensitivity, and resolution. It concludes that the proposed sensor exhibits superior wavelength sensitivity compared to others documented in the literature and demonstrates the potential of ML to elevate optical sensor design.

**Table 3:** Comparative Studies of Performance Analysis.

Ref.	RI	*S*_*λ*_	*R*	ML
		(nm/RIU)	(RIU)	
[12] (2024)	1.29-1.36	10,000	–	ML
[36] (2024)	1.31-1.40	18,000	–	ML
[37] (2020)	1.33-1.42	21,000	4.76 × 10^−6^	–
[38] (2022)	1.35-1.41	8,400	2.13 × 10^−5^	–
[39] (2020)	1.33-1.40	10,700	9.34 × 10^−6^	–
[40] (2020)	1.33-1.44	11,200	8.92 × 10^−6^	–
This work (2024)	1.34-1.41	30,000	3.33 × 10^−6^	ML

### 3.3 Consideration of experiment setup

**Figure 7:**
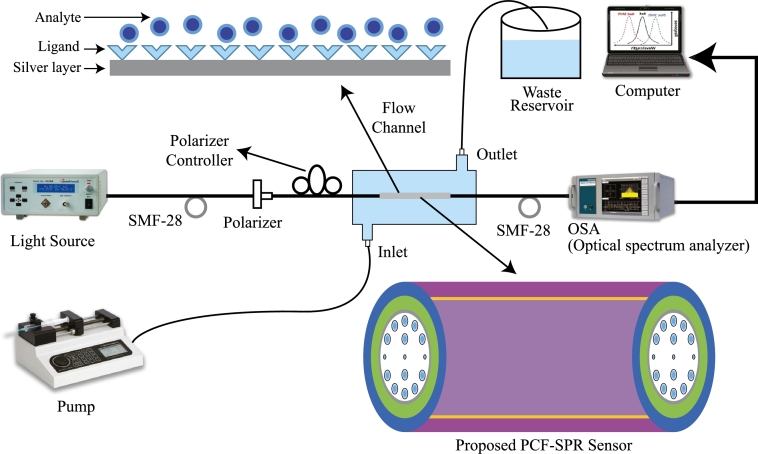
The experimental setup of the proposed sensor for practical sensing purposes.

Fig. 7 depicts the experimental setup for the plasmonic sensor, carefully engineered to ensure precise and efficient data capture. The system initiates with a supercontinuum light source, crucial for its broad spectral coverage from 450 to 1600 nm. The SuperK model from NKTPhotonics™ [41], chosen for its wide range and stability, is ideal for sensing applications that demand consistent light properties across a diverse spectrum. Before the light reaches the sensor, it undergoes polarization to maintain a specific orientation of its electric field, a key factor for the precise interactions required in plasmonic sensing. The polarized light is then channeled through a single-mode fiber, specifically the SMF-28 type, selected for its capability to maintain the integrity of the light signal by allowing only one mode of light to propagate. This minimizes signal distortion and loss.

As the light passes through the initial SMF, it enters the plasmonic sensor, interacting with its properties and altering the light based on the external conditions the sensor is designed to detect. Once the light exits the sensor, it is directed through another SMF to an optical spectrum analyzer, specifically the Yokokawa™ AQ6370C model. This analyzer examines the light spectrum post-interaction, providing critical data on how the sensor environment influenced the light. Connections between the sensor and the SMFs are established using filament fusion splicing, utilizing the Vytran FFS-2000 splicer [42]. This method ensures robust, high-quality connections with minimal loss at the junction points. Precision alignment techniques ensure proper SMF and PCF connectivity. This alignment is vital for effective light transfer and accurate sensor readings. Additionally, the setup investigates alternative coupling methods, such as using an etched SMF tip or specialized couplers, to potentially enhance coupling efficiency, which has been recorded to range between 80% and 90%.

The insertion of an analyte conduit enables the introduction of a liquid analyte intake and discharge mechanism at an appropriate location. The analyte is introduced into the conduit using a meticulously programmed microinjection pump (LSP01-1A, LongerPump™). The utilized analyte is then stored within a waste reservoir linked to the conduit's outlet. The resonance wavelength undergoes modifications, shifting towards higher or lower wavelengths in the presence of numerous unidentified analytes. These spectral variations can be detected using an optical spectrum analyzer. The ultimate step entails employing a computer to assess the peak wavelength shifts and exhibit the conclusive SPR output spectra.

### 3.4 Performance analysis using ML

#### 3.4.1 Performance metrics

Table 4 presents the 10-fold dataset results for the MLR model of confinement loss. The overall MAE, MSE, RMSE, and R squared values of the MLR model are 5.22, 121.93, 9.48, and 0.96, respectively.

**Table 4:** Performance analysis of MLR for confinement loss prediction.

Fold	MAE	MSE	RMSE	R squared value
1	3.27	15.20	3.89	0.96
2	8.15	336.64	18.34	0.94
3	6.23	221.65	14.88	0.94
4	3.84	26.99	5.19	0.97
5	4.87	51.28	7.16	0.97
6	4.40	33.46	5.78	0.96
7	8.34	370.65	19.25	0.91
8	3.69	20.83	4.56	0.97
9	5.73	119.56	10.93	0.96
10	3.69	23.01	4.79	0.98
Average	5.22	121.93	9.48	0.96

Table 5 presents the 10-fold dataset results for the SVR model of confinement loss, showing that the overall MAE, MSE, RMSE, and R-squared values are 23.19, 2769.74, 48.52, and -0.08, respectively.

**Table 5:** Performance analysis of SVR for confinement loss prediction.

Fold	MAE	MSE	RMSE	R squared value
1	12.73	455.36	21.33	-0.05
2	36.29	6834.13	82.66	-0.12
3	26.47	4575.06	67.63	-0.05
4	17.00	1020.04	31.93	-0.09
5	23.92	2652.82	51.50	-0.08
6	16.70	942.54	30.70	-0.07
7	35.60	5179.27	71.96	-0.16
8	15.78	844.41	29.05	-0.02
9	28.76	3904.77	62.48	-0.11
10	18.63	1288.96	35.90	-0.10
Average	23.19	2769.74	48.52	-0.08

Table 6 displays the 10-fold evaluation results of the dataset using the RFR model of confinement loss. The overall MAE, MSE, RMSE, and R-squared values for the RFR model are 3.54, 160.33, 10.15, and 0.95, respectively.

**Table 6:** Performance analysis of RFR for confinement loss prediction.

Fold	MAE	MSE	RMSE	R squared value
1	0.99	5.10	2.26	0.98
2	8.74	574.67	23.97	0.90
3	4.63	271.40	16.47	0.93
4	2.09	35.42	5.95	0.96
5	4.11	172.87	13.14	0.92
6	2.01	35.33	5.94	0.95
7	7.15	444.94	21.09	0.90
8	2.26	30.67	5.53	0.96
9	2.50	30.20	5.49	0.99
10	0.90	2.68	1.63	0.99
Average	3.54	160.33	10.15	0.95

Table 7 displays the 10-fold cross-validation results of the dataset for the MLR model for wavelength. The overall values for MAE, MSE, RMSE, and R-squared are 0.09, 0.01, 0.11, and 0.48, respectively.

**Table 7:** Performance analysis of MLR models for wavelength loss prediction.

Fold	MAE	MSE	RMSE	R squared value
1	0.10	0.01	0.11	0.36
2	0.11	0.02	0.14	0.31
3	0.09	0.02	0.13	0.50
4	0.10	0.02	0.12	0.45
5	0.07	0.01	0.08	0.78
6	0.11	0.02	0.13	0.50
7	0.09	0.01	0.12	0.42
8	0.09	0.01	0.11	0.58
9	0.08	0.01	0.10	0.56
10	0.11	0.02	0.13	0.40
Average	0.09	0.01	0.11	0.48

Table 8 displays the 10-fold evaluation results of the dataset for the SVR model. The SVR model's overall metrics include an MAE of 0.11, an MSE of 0.02, an RMSE of 0.14, and an R-squared value of 0.24.

**Table 8:** Performance analysis of SVR models for wavelength prediction.

Fold	MAE	MSE	RMSE	R squared value
1	0.09	0.01	0.11	0.44
2	0.15	0.03	0.18	-0.17
3	0.13	0.03	0.17	0.14
4	0.11	0.02	0.13	0.44
5	0.12	0.02	0.14	0.38
6	0.13	0.02	0.16	0.26
7	0.10	0.01	0.12	0.40
8	0.09	0.01	0.11	0.59
9	0.11	0.02	0.14	0.21
10	0.16	0.03	0.19	-0.22
Average	0.11	0.02	0.14	0.24

Table 9 shows the 10-fold evaluation results of the dataset for the RFR model. The overall metrics for the RFR model are an MAE of 0.01, an MSE of 0.001, an RMSE of 0.03, and an R-squared value of 0.96.

**Table 9:** Performance analysis of RFR models for wavelength prediction.

Fold	MAE	MSE	RMSE	R squared value
1	0.01	0.001	0.02	0.97
2	0.02	0.003	0.05	0.90
3	0.01	0	0.01	0.99
4	0.01	0.002	0.04	0.93
5	0.02	0.002	0.05	0.92
6	0.01	0.001	0.03	0.97
7	0.01	0	0.02	0.99
8	0.01	0.001	0.02	0.98
9	0.01	0	0.01	1.00
10	0.01	0.001	0.03	0.97
Average	0.01	0.001	0.03	0.96

#### 3.4.2 Predictions using ML algorithm

**Figure 8:**
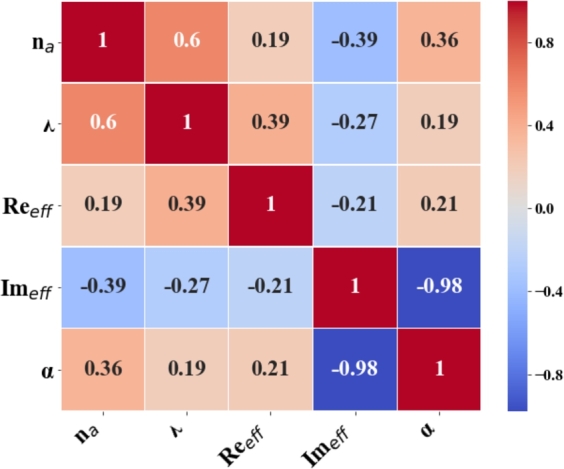
Feature variables of a PCF-SPR sensor using a heatmap.

Fig. 8 depicts the heatmap visualizes the correlation coefficients between five variables related to a plasmonic sensor: refractive index ($n_{a}$), wavelength (*λ*), the real part of the effective refractive index ($Re_{\text{eff}}$), the imaginary part of the effective refractive index (${\text{Im}}_{\text{eff}}$), and the confinement loss (*α*). Each cell in the heatmap represents the correlation between the pair of variables corresponding to its row and column, with the color indicating the strength and direction of the correlation. The diagonal cells indicate a perfect positive correlation of each variable with itself, always represented by the darkest red color. The correlation coefficient between $n_{a}$ and *λ* is 0.6, indicating a moderately positive correlation. As $n_{a}$ increases, the *λ* also tends to increase. The correlation coefficient between $n_{a}$ and ($Re_{\text{eff}}$) is 0.19, indicating a weak positive correlation. The $n_{a}$ has a slight positive relationship with the ($Re_{\text{eff}}$).

The correlation coefficient between $n_{a}$ and ${\text{Im}}_{\text{eff}}$ is -0.39, indicating a moderately negative correlation. As $n_{a}$ increases, ${\text{Im}}_{\text{eff}}$ tends to decrease. The correlation coefficient between $n_{a}$ and *α* is 0.36, indicating a moderately positive correlation between $n_{a}$ and *α*. The correlation coefficient between *λ* and $R_{\text{eff}}$ is 0.39, indicating a moderately positive correlation. As *λ* increases, $R_{\text{eff}}$ also tends to increase. The correlation coefficient between *λ* and ${\text{Im}}_{\text{eff}}$ is -0.27, indicating a weak negative correlation. As *λ* increases, ${\text{Im}}_{\text{eff}}$ tends to decrease. Finally, the correlation coefficient between *λ* and *α* is 0.19, indicating a weak positive correlation between *λ* and *α*.

The correlation coefficient between $R_{\text{eff}}$ and ${\text{Im}}_{\text{eff}}$ is -0.21, indicating a weak negative correlation. As $R_{\text{eff}}$ increases, the imaginary part ${\text{Im}}_{\text{eff}}$ tends to decrease slightly. The correlation coefficient between $R_{\text{eff}}$ and *α* is 0.21, indicating a weak positive correlation between $R_{\text{eff}}$ and *α*. The correlation coefficient between ${\text{Im}}_{\text{eff}}$ and *α* is -0.98, indicating a robust negative correlation. This means that as the imaginary part of the effective refractive index ${\text{Im}}_{\text{eff}}$ increases, the confinement loss *α* decreases significantly. The color scale on the right indicates the strength and direction of the correlations. Dark red represents strong positive correlations, dark blue represents strong negative correlations, and lighter colors represent weaker correlations. The value range is from -1 (perfect negative correlation) to 1 (perfect positive correlation). In machine learning, understanding these correlations is crucial for feature selection and relationships between variables. Strong correlations might indicate redundant features, while weak correlations might suggest independent features that could provide unique information to the model.

**Figure 9:**
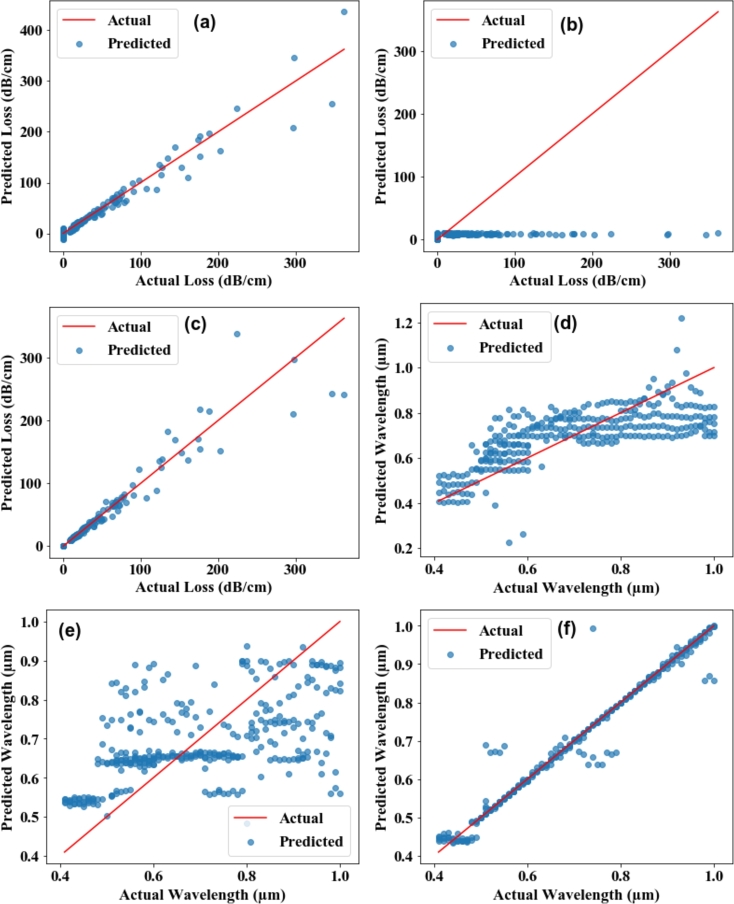
Confinement loss prediction using (a) MLR, (b) SVR, and (c) RFR algorithm. Wavelength prediction using (a) MLR, (b) SVR, and (c) RFR algorithm.

Fig. 9 (a) shows the relationship between the simulated confinement loss (dB/cm) from a plasmonic sensor and the predicted confinement loss (dB/cm) from an MLR model. The close alignment of many blue dots with the red line indicates that the MLR model performs well in predicting confinement loss within a specific range. However, a few data points significantly deviate from the red line, particularly at higher values of confinement loss. These outliers suggest areas where the model's predictions are less accurate. The model accurately predicts lower and mid-range confinement loss values, as evident from the dense clustering of blue dots along the red line in these regions. However, accuracy decreases for higher confinement loss values, with the increased dispersion of blue dots away from the red line indicating this trend. The figure illustrates that the MLR model is a robust tool for predicting confinement loss in plasmonic sensors, demonstrating high accuracy for lower and mid-range values.

Fig. 9 (b) illustrates the relationship between the simulated confinement loss (dB/cm) obtained from a PCF-SPR sensor and the predicted confinement loss (dB/cm) derived from the SVR model. This analysis aims to assess the performance of the SVR model in predicting confinement loss by examining the correlation between the actual and predicted values. The close clustering of blue dots near the x-axis indicates that the SVR model consistently underpredicts the confinement loss across the range of actual values. The lack of dispersion in the predicted values, with most being close to zero, suggests that the SVR model is not effectively capturing the variability in the actual confinement loss data. The SVR model shows poor accuracy in predicting lower and mid-range confinement loss values, as evidenced by the blue dots' significant deviation from the red line. The model also fails to accurately predict higher confinement loss values, with no clear pattern of correct prediction at these levels. The figure demonstrates that the SVR model is currently inadequate for predicting confinement loss in PCF-SPR sensors, with significant underprediction across the range of actual values.

Fig. 9(c) illustrates the relationship between the simulated confinement loss (dB/cm) obtained from a plasmonic sensor and the predicted confinement loss (dB/cm) derived from the RFR model. The close alignment of many blue dots with the red line indicates that the RFR model performs well in predicting confinement loss within a specific range. However, several data points significantly deviate from the red line, particularly at higher values of confinement loss. These outliers suggest areas where the model's predictions are less accurate. The model is highly accurate in predicting lower and mid-range confinement loss values, as evidenced by the dense clustering of blue dots along the red line in these regions. However, the accuracy decreases for higher confinement loss values, as indicated by the increased dispersion of blue dots away from the red line. The figure demonstrates that the RFR model is a robust tool for predicting confinement loss in PCF-SPR sensors, with high accuracy for lower and mid-range values.

Fig. 9 (d) illustrates the MLR model's performance in predicting the plasmonic sensor's wavelength (μm). The overall trend of the blue dots following the red line suggests that the MLR model captures the general relationship between the actual and predicted wavelengths. However, the presence of deviations and spread around the red line indicates that, while the MLR model performs reasonably well, there are instances of both underestimation and overestimation. All blue dots would lie exactly on the red line in a perfectly accurate and precise model. The scatter observed indicates that the model has a degree of error and variance in its predictions. The clustering of data points closer to the red line in certain regions (e.g., 0.4 to 0.6 micrometers) demonstrates better prediction performance in these ranges. In contrast, the more extensive spread in the other areas indicates less reliability. Overall, the provided figure showcases the MLR model's effectiveness in predicting the wavelength values of the PCF-SPR sensor.

The provided Fig. 9(e) illustrates the performance of an SVR model in predicting a plasmonic sensor's wavelength (μm). Below is an analytical analysis of the model's performance based on the data depicted in the scatter plot. All blue dots would lie exactly on the red line in a perfectly accurate and precise model. However, the significant scatter of blue dots around the red line suggests considerable prediction error and variance. This observed pattern indicates that the SVR model struggles to accurately predict the wavelengths, with substantial instances of underestimation and overestimation. The SVR model exhibits significant prediction errors and variance in predicting the wavelength values of the plasmonic sensor. The model shows a considerable deviation from the actual wavelength values, indicating areas where accuracy and precision need substantial improvement.

The provided Fig. 9 (f) illustrates the performance of the RFR model in predicting the wavelength (μm) of a plasmonic sensor. All blue dots would lie exactly on the red line in a perfectly accurate and precise model. The closer the blue dots are to the red line, the higher the model's accuracy and precision. The observed scatter of blue dots around the red line indicates a degree of prediction error and variance. This spread suggests that while the RFR model is generally accurate, it is not perfectly precise, with instances of both underestimation and overestimation. The random forest regression model demonstrates a strong capability in predicting the wavelength values of the plasmonic sensor, especially within specific wavelength ranges. While the model effectively captures the general trend, it exhibits some error and variance, indicating areas where accuracy and precision can be improved.

## 4 Conclusions

The study recorded a series of observations according to its findings. Firstly, silver coatings applied to the sensor significantly contribute to its performance, and the thickness of these coatings plays a crucial role. The study recorded a series of observations according to its findings. Firstly, silver coatings applied to the sensor significantly contribute to its performance, and the thickness of these coatings plays a crucial role. The comprehensive numerical simulations reveal that the sensor exhibits a $3.33\times 10^{-6}$ RIU resolution, attaining a maximum wavelength sensitivity of 30,000 nm/RIU. The sensor's heightened sensitivity is particularly noteworthy within the RI detection range, from 1.34 to 1.41. Secondly, the research utilizes ML algorithms such as MLR, SVR, and RFR to forecast confinement loss and wavelength for different analytes. The fabrication process for this sensor is straightforward, leveraging existing technology, and its symmetrical structure enhances its practicality. The presented sensor holds substantial promise for biomedical and chemical research applications, offering precise and accurate detection capabilities suitable for deployment in bio-sensing applications.

Future work in this area could focus on experimental validation of the sensor's performance and its application in real-world scenarios. Incorporating an innovative deep learning method enhanced the sensor's precision and minimized errors, opening avenues for groundbreaking advancements across various applications, such as lab-on-chip technologies.

## Funding

There are no funds for this research.

## CRediT authorship contribution statement

**Chanchal Saha:** Writing – original draft, Investigation, Formal analysis, Data curation. **Farzana Haque:** Writing – original draft, Methodology, Data curation, Conceptualization. **Nazrul Islam:** Validation, Formal analysis, Conceptualization. **Muhammad Minoar Hossain:** Resources, Formal analysis. **Md. Easin Arafat:** Validation, Methodology. **Mohammad Abu Yousuf:** Supervision. **Mohammad Motiur Rahman:** Supervision.

## Declaration of Competing Interest

The authors declare that they have no known competing financial interests or personal relationships that could have appeared to influence the work reported in this paper.

## Data Availability

The corresponding author will provide access to the data and materials upon request.
